# Extreme Temperatures and Firm-Level Stock Returns

**DOI:** 10.3390/ijerph18042004

**Published:** 2021-02-19

**Authors:** Jingbin He, Xinru Ma

**Affiliations:** School of Management and Economics, University of Electronic Science and Technology of China, Chengdu 611731, China; hejingbin@foxmail.com

**Keywords:** extreme temperature, stock return, investor mood

## Abstract

By linking stock returns with weather conditions from 2007 to 2019 in China, we study how firm-level stock returns react to extreme temperatures. Based on a multivariate ordinary least squares regression model with fixed effects, empirical results show that firm-level stock returns decrease with exposure to extreme temperatures. We further explore the heterogeneity in the temperature-return relation to enrich our understanding of the economic mechanism behind it. The impact of extreme temperatures on abnormal stock returns is more pronounced in smaller, younger, more volatile, less profitable firms and firms with more intangible assets. The results indicate that the investor mood likely plays a role in the extreme temperature effect. The impact of extreme temperatures holds after addressing a series of concerns. Overall, our paper provides additional firm-level evidence on the environment-induced mood effect in the stock market.

## 1. Introduction

A large strand of scientific evidence overwhelmingly supports climate change. It is well recognized that our climate is nonstationary [1], and extreme weather and climate events are becoming more prevalent in recent decades [2]. Specifically, the frequency of heatwaves has more than doubled in some locations [3], and winter storms have also become more severe in frequency and intensity [4].

In the past decades, the alarming facts on climate change have drawn the attention of economists. For instance, it has been found that temperature has significant and systematic effects on economic outcomes, such as income [5], economic growth [6,7], economic production [8], and professional decision-making [9]. As for the effect of temperature on the stock market, previous studies in the literature have observed that temperature is associated with aggregated stock market returns [10,11]. However, whether and how temperature affects firm-specific stock performance remains an underexplored research question.

In this paper, we investigate the link between the temperature of listed firms’ locations and their stock performance from a relatively micro perspective. Some existing literature has provided several insights on this topic. Bansal et al. [12] propose a model of temperature-induced disasters and document that temperature shock has a negative effect on stock prices. Balvers, Du and Zhao [11] find that the risk premium on temperature shock is significantly negative by analyzing temperature in an Arbitrage Pricing Theory model. Notably, the foundation of this association is the impact of temperature on mood. Howarth and Hoffman [13] show that temperature is one of the three factors that exert the greatest influence on mood. Psychological evidence also indicates that temperature extremes may lead to apathy [14] and aggression [13]. Besides, numerous studies in the literature indicate that unpleasant ambient conditions significantly decrease the stock market performance, presumably due to depressed mood [15,16]. Motivated by these findings, we conjecture that the extreme temperature may result in subpar stock performance.

To test our conjecture, we link listed firms’ stock returns in the Chinese stock market with the temperature related to their headquarter locations. The Chinese stock market is a suitable setting to test our conjecture for several reasons. First, China is a vast territory, and each city’s temperature is not synchronized with the temperature in other cities, which provides enough variation among the temperatures of listed firms’ locations. Second, the Chinese stock market adopts a full electronic order-driven trading system without market makers; that is, all orders submitted by investors are directly entered into the matching system of the exchange through brokers. Thus, any concern about the potentially contaminating effects of market makers could be mitigated. Third, individual investors in the Chinese stock market play a dominant role. The majority of the total stock market value is taken by individual investors [17]. According to the 2017 Annual Statistics of the Shanghai Stock Exchange, individual trading accounted for 82.01% of the total market trading volume [18]. Most individual investors in the Chinese stock market are short-term speculators [19]. Besides, relative to institutional investors, individual investors are less informed and more vulnerable to be affected by psychological biases [20,21,22,23]. Hence, if the impact of temperature-induced mood exists, it should manifest in our setting.

We begin by examining the relation between extreme temperature and stock performance, using the abnormal stock returns of all firms listed in China from 2007 to 2019. We link firm-specific abnormal stock returns with the temperatures related to the firm’s location. Using a multivariate ordinary least squares regression model with fixed effects, we find that the extreme temperature is negatively associated with the contemporaneous abnormal return. Even after we control for a series of weather conditions and rule out the seasonal effects, this finding remains robust. The extreme temperature effect is also economically meaningful that a one-standard-deviation increase in extreme temperature will reduce the abnormal stock return of approximately 2–3% per year. Thus, the result verifies our conjecture that extreme temperature induces depressive mood and leads to low abnormal stock return.

Next, we explore the heterogeneity in the relation between extreme temperature and abnormal stock return to deepen our understanding of the underlying mechanism. Baker and Wurgler [24] demonstrate the link between investors’ mood and stock performance. Specifically, they show that firms that are hard to value and arbitrage are more sensitive to the fluctuations in mood. Based on the firm characteristics suggested by Baker and Wurgler [24], empirical results show that the temperature-return relation is more pronounced in firms with smaller size, younger age, higher volatility, lower profitability, and higher intangible asset ratio. We also put all the interaction terms in an overarching regression model and find that a large proportion of those interaction terms remains significant. Overall, our analyses are consistent with Baker and Wurgler [24]’s view and provide robust and concrete evidence that mood is a mediator.

We then explore whether there are any other mediators besides mood. Motivated by the previous literature [25], we first investigate whether productivity is a mediator but find little evidence to support that extreme temperature affects labor productivity. We next focus on the temperature-induced disasters and find that the relation between extreme temperatures and stock returns for steel and construction firms is insignificant. This result can be explained by the trade-off between two competing temperature effects. Specifically, this result implies that temperature-induced disasters might provide profit-making opportunities for steel and construction firms to benefit from rebuilding the city. Thus, the negative effect of extreme temperatures on stock returns through mood might be offset by this profit-making effect for steel and construction firms.

Besides, there still remain several concerns. One concern is that our findings may be driven by agricultural firms. Intuitively, the extreme temperature may harm agricultural production. Hence, investors convey this negative information into the stock market, shown as low abnormal stock returns. To mitigate this concern, we exclude all firms in the industry of agriculture, forestry, stock raising, and fishing and find that the effect of extreme temperature is still robust in the subsample. Another concern is that the findings might be driven by events. For example, Christophe et al. [26] and Park et al. [27] show that the stock market reacts to earnings announcements significantly. Besides, the previous literature shows that the market reacts negatively to Mergers and Acquisitions (M&A) announcements [28,29]. To rule out this potential concern, we exclude months in which firms issue quarterly reports, semi-annual reports, or annual reports. Additionally, we exclude observations if M&A announcements are available in the current month or subsequent 6 months. We find that the extreme temperature effect remains robust, suggesting that our main finding is not merely due to major events.

To verify our robustness, we also examine the sensitivity of our results to the matching method of weather stations, the formation periods, and asset pricing models. We first use the average temperature within a radius from the firm location as a robust approach and find that our main finding is still robust. According to Fama and French [30], we then use the stock returns of the past 36, 48, and 60 months to estimate βs from Capital Asset Pricing Model (CAPM) regressions. We find that the results under different formation periods are consistent with our main finding. Additionally, we also use the Fama–French three-factor (FF3) model [31] and Fama–French five-factor (FF5) model [32] to estimate the expected stock return and find that the extreme temperature effect remains robust. Overall, the negative relation between extreme temperature and abnormal return is not sensitive to the formation periods and asset pricing models we choose to adjust stock raw return.

Finally, we investigate whether the extremely high and low temperatures have different impacts on abnormal stock return. Previous studies in psychology [13,33,34] show that a lower temperature is related to aggression (e.g., risk-taking), and a higher temperature is associated with both aggression and apathy (e.g., risk aversion), indicating that high temperature and low temperature might have different effects. Motivated by these findings, we split extreme temperatures into extremely high temperatures and extremely low temperatures. We find that two variables are both negatively related to the abnormal stock return. Taken together, the result shows that both extremely high and low temperatures will induce a depressed mood and lead to negative stock performance.

This paper contributes to the climate economy literature in several ways. First, a rapidly growing literature has shown that temperatures affect various economic outcomes at the aggregate level [5,7,8]. For example, the literature on the stock market studies the effect of temperature on the market returns [10] and systematic risk [11]. However, little literature explores the effect of temperature at a more micro-firm level. We fill this gap by linking firm-level stock returns with the local temperatures in China.

Second, the climate economy literature explores the effect of extreme temperatures on labor supply [35], agricultural outcomes [36,37], and productivity [6,38]. Only a few recent papers study the effects of climate shocks in financial markets from the angle of market efficiency [39], systematic risk [11], and sales of publicly listed firms [25]. However, the extent to which extreme temperatures affect investor mood remains an underexplored research question. To the best of our knowledge, we are the first study to examine how extreme temperatures affect stock returns.

Third, the previous literature on climate-induced mood in the stock market verifies their conjecture based on the baseline relation between climate conditions and stock returns [16,40]. However, they do not investigate the heterogeneity in the baseline relation, which is a more reasonable research design to explore the potential channel. Using the firm characteristics suggested by Baker and Wurgler [24], we verify that our temperature-return relation is more pronounced in firms whose performance is more likely to be affected by investor sentiment. Thus, our heterogeneity analyses shed light on the conditions that can accentuate or attenuate the biases induced by ambient circumstances. Besides, our study also provides new evidence to support the findings of Baker and Wurgler [24].

The rest of the paper is organized as follows. Section 2 describes the materials and methods. Section 3 analyzes the results of how temperature extremes affect firm-specific stock performance. Section 4 is the discussion. Section 5 concludes.

## 2. Materials and Methods

### 2.1. Sample and Stock Performance

Our data about stock returns are obtained from China Stock Market and Accounting Research (CSMAR). Based on the fact that the split share structure reform in China was basically completed at the end of 2006, we choose our sample from January 2007 to December 2019. Following the literature convention [41,42], we exclude financial firms, ST (special treatment) firms, and PT (particular transfer) firms from our sample as they are specially regulated due to financial distress. The first-day return is extremely high in China. Song et al. [43] investigate 948 Chinese firms and find that the average initial returns are 66%. Therefore, we eliminate the first-month data for new Initial Public Offering (IPO) firms as they behave differently in other time periods in China.

We then describe our main dependent variable (*Ret_Abnormal*), which is each firm’s monthly abnormal return and is calculated as follows:

(1) $$Ret\_Abnormal_{i,t}=Ret\_Raw_{i,t}-Ret\_Bench_{i,t}$$

where $Ret\_Abnormal_{i,t}$ is the abnormal stock return of firm *i* in month *t*, $Ret\_Raw_{i,j}$ is the raw stock return of firm *i* in month *t*, and $Ret\_Bench_{i,t}$ is the expected return of firm *i* in month *t* and is calculated by the CAPM model as follows:

(2) $$Ret\_Bench_{i,t}=\beta_{i,t}*\left(Rm_{t}-Rf_{t}\right)+Rf_{t}$$

where $Rm_{t}$ is the market return in month *t*, and $Rf_{t}$ is the contemporaneous risk-free rate. We follow the literature convention to use the one-year deposit rate as the risk-free rate [44,45]. We obtain the data from the CSMAR. $\beta_{i,t}$ is estimated from the following CAPM regression, using data in the recent two years that the estimating month τ∈(t−24,t−1):

(3) $$Ret\_Raw_{i,\tau }-Rf_{\tau }=\alpha_{i,t}+\beta_{i,t}*\left(Rm_{\tau }-Rf_{\tau }\right)+\varepsilon_{i,\tau }$$

where $Ret\_Raw_{i,t}$ is the raw stock return of firm *i* in month τ, $Rm_{\tau }$ is the market return in month τ, and $Rf_{\tau }$ is the contemporaneous risk-free rate.

### 2.2. Weather Conditions

The weather data are from the official website of the China Meteorological Administration. They disclose the longitude and latitude of each weather station and daily weather conditions, including temperature, rainfall, sunshine duration, wind speed, humidity, and air pressure. To match listed firms with related weather conditions, we obtain the geographic location (i.e., the longitude and latitude) of each listed firm’s annual headquarter address from the CSMAR. Based on the haversine distance formula, we then calculate the distance between each weather station and each firm’s headquarter address in a given year. Next, we match each firm with the weather conditions of the nearest weather station for each year [46]. Using this method, we can build a one-to-one correspondence between cities and observation stations annually. Some previous literature matches firm locations with the weather conditions by city names [47]. However, the matched weather conditions might be biased if one city has no station or multiple weather stations. Our method based on the haversine distance formula can address this issue.

We next turn to describe the variables of interest in our paper. In the spirit of Addoum, Ng, and Ortiz-Bobea [25], *ExtremeTemperature* is defined as the proportion of days with extreme temperatures in a month. Specifically, we define a day’s temperature as extreme if its daily highest temperature is over 30 °C or lowest temperature is below 0 °C to explore whether extremely high and low temperatures affect mood differently, we also calculate the proportion of days with extremely high temperatures and low temperatures separately.

One important factor that might bias our baseline estimator of temperature extremes is seasonal affective disorder (SAD), a sub-type of recurrent mood disorder with a characteristic pattern of onset and remission. Kamstra et al. [48] have shown that SAD significantly affects the stock market returns. To construct SAD, we first calculate the night duration. As the location addresses are all in the northern hemisphere, the night duration is calculated as follows:

(4) $$H_{i,t}=24-7.72*arccos\left[-\tan\left(\frac{2\pi \delta_{i,t}}{360}\right)*\tan\left(\gamma_{t}\right)\right]$$

where $H_{i,t}$ is the night duration in hours. $\delta_{i,t}$ is the latitude of firm *i*’s headquarter address in month *t*; $\gamma_{t}$ is the sun’s declination angle, which equals $0.4102*\sin\left[\left(\frac{2\pi }{365}\right)*\left(julian_{t}-80.25\right)\right]$; and $julian_{t}$ equals 1 for 1 January, 2 for 2 January, and so on in each year. Specifically, we choose the 15th day of month *t* to measure $julian_{t}$. To consider the night duration in all seasons, we do not replace the values with zero in spring and summer.

In the spirit of Kamstra, Kramer, and Levi [48], we measure *SAD* as follows:

(5) $$SAD_{i,t}=H_{i,t}-12$$

where $SAD_{i,t}$ is the SAD of firm *i* in month *t*.

The previous literature has verified that stock markets can be affected by other weather conditions, such as cloud cover [16,49,50], wind speed [51], and humidity [52]. Therefore, we construct several variables to control for these weather conditions. First, as the China Meteorological Administration does not disclose cloud cover, we construct two other variables to capture the cloud cover effect. The first one is *Sunshine*, which equals the average sunshine duration in each month for each firm’s headquarter address. Sunshine duration is considered significantly related to cloud cover as the least cloudy areas are sunniest and vice versa [53]. Xia [54] also demonstrates the inverse relation between sunshine duration and cloud cover. The second one is *Rain*, which equals the fraction of rainy days in each month for each firm’s headquarter address. The literature shows that rainfall is also positively correlated with cloud cover [55,56]. Next, we construct *Wind*, which equals the fraction of strong wind days in each month for each firm’s headquarter address, to control wind speed. As Lu and Chou [40] documented, people do not generally feel the wind effect when its speed is less than 5 km/h. Hence, we define a day as a strong windy day if the daily wind speed is higher than 5 km/h. Besides, we also control for the average relative humidity (*Humidity*) and the average air pressure (*AirPressure*) in each month for each firm’s headquarter address. We present the variables related to the weather conditions in Table 1 to facilitate reading.

### 2.3. Summary Statistics

Table 2 shows the summary statistics for the full sample used for analysis. We winsorize all variables at 1% in both tails, which is widely used to mitigate the influence of outliers [57,58,59]. On average, the abnormal stock return equals 0.404% (*Ret_Abnormal*). As for the variable of interest, *ExtremeTemperature*, we find 36.1% of a month experiences extreme temperature. In some months, all (no) days are of extreme temperature as the 95th (5th) percentile equals one (zero). The mean value of *SAD* is −0.117, indicating that the night duration in our sample period is generally less than 12 h. On average, the sunshine ratio per day equals 0.219, compared with 24 h per day. About 31.8% of a month contains rainy days, and 78.4% of a month is windy outside. As for humidity, the mean value is 0.699. The air pressure in our sample period is 0.995 on average. It suggests that the mean value of the air pressure is almost equal to one standard atmospheric pressure. Overall, the statistics show that our sample is well balanced.

## 3. Results

### 3.1. Baseline Results

In this section, we assess whether extreme temperatures influence the stock returns. When temperatures deviate from the best ambient temperature, it tends to mediate people’s emotions [10,60,61]. The Centers for Disease Control and Prevention (CDC of the US) also states that the rise in high temperature indicates the potential impacts on depression [62]. Hence, we expect that the stock return will be lower when the local extreme temperature is larger. Specifically, the regression is as follows:

(6) $$Ret\_Abnormal_{i,t}=\alpha +\beta_{1}\times ExtremeTemperature_{i,t}+\sum (\gamma_{n}\times Controls_{n,i,t})+\varepsilon_{i,t}$$

where $Ret\_Abnormal_{i,t}$ is the abnormal return of firm *i* in month *t*. Details of constructing the abnormal return are described in Section 2.1. $ExtremeTemperature_{i,t}$ is the fraction of days with extreme temperature in month *t* for firm *i*’s headquarter location. $Controls_{i,t}$ are control variables, including the lagged abnormal return of firm *i* in month *t*−1 (*Lagged_Ret_Abnormal*) and other weather condition controls defined in Table 1. Following Wu, Hao, and Lu [47], we include stock fixed effects to control for the unobservable firm-level factors (e.g., financial conditions and fundamentals) and include month fixed effects to control for the seasonal effects that might bias our results. Standard errors are clustered at the firm level and reported in parentheses below the regression coefficients. We use ***, **, and * to denote significance at the 1%, 5%, and 10% level (two-sided), respectively. All the following regression results are reported in the same way.

Results are shown in Table 3. Column 1 shows the relation between *ExtremeTemperature* and *Ret_Abnormal* with no specific controls. The significant negative coefficient on *ExtremeTemperature* indicates that a larger extreme temperature experienced by the investors is associated with a lower firm-specific stock return. In Column 2, we include a series of controls to mitigate the estimating bias driven by the omitted variable problem. First, we include past stock return (*Lagged_Ret_Abnormal*) to control for the momentum. Second, as the previous literature shows that SAD significantly affects stock market returns [48], we additionally include *SAD* as a control variable. Third, several weather proxies that have been shown to affect stock returns significantly are included in the regression, such as *Sunshine*, *Rain*, *Wind*, *Humidity*, and *AirPressure*. The coefficient of *ExtremeTemperature* in Column 2 is −0.695 and still significant at the 1% level. This result suggests that a one-standard-deviation increase in *ExtremeTemperature* will reduce the abnormal stock return by approximately 0.244% per month, or 2.927% per year.

Another concern is that our results might be driven by unobservable annual factors. For example, the stock returns might be lower as the Chinese stock market becomes mature, indicating that stock returns decrease over the years. As climate scientists document extreme weather conditions increases over the years [3], the negative relation between extreme temperature and stock returns is just because both of them vary from year to year. To mitigate this concern, we additionally include year fixed effects in Columns 3 and 4. We find that the coefficients are still negative and significant at the 1% level whether we add control variables or not. Our conjecture is formally verified in Column 4. The coefficient of *ExtremeTemperature* in Column 4 equals −0.500, indicating that a one-standard-deviation increase in temperature extremes will reduce the abnormal stock return by 0.176% per month, or 2.106% per year. 

The empirical results show that a larger proportion of extreme temperature in a month is associated with a lower stock return. It is consistent with our conjecture. When the ambient environment is extremely cold or hot, investors’ mood tends to be depressed. Consequently, the stock experiences a low return.

### 3.2. Does Extreme Temperature Affect Stock Market Returns through Mood as the Mediator?

We now turn to explore the heterogeneity in the relation between extreme temperature and the stock return. Baker and Wurgler [24] demonstrate that the stock returns are more likely to be influenced by investors’ mood for firms whose valuations are highly subjective and difficult to arbitrage. Hence, if our results are truly driven by mood, we expect to observe more pronounced results in certain firms indicated by Baker and Wurgler [24]. Three aspects of firm characteristics related to firms’ valuation suggested by Baker and Wurgler [24] are size and age, profitability, and asset tangibility.

#### 3.2.1. Size and Age Characteristics

In this category of firm characteristics, Baker and Wurgler [24] focus on firm size, age, and volatility. Intuitively, smaller, younger, and more volatile firms are more difficult to value. We begin by exploring whether the relation between extreme temperature and stock return differentiates across firm size, age, and volatility. For each baseline regression model based on Equation (6), we add an interaction variable equal to the product of *ExtremeTemperature* and one of the size and age characteristics.

Results are reported in Table 4. For conciseness, we include control variables but do not report the coefficients of control variables. The coefficients are consistent with those in baseline results. In Panel A, we construct the interaction term between extreme temperature and normalized firm size, which equals the natural logarithm of book equity in the recent fiscal year (*LogFirmSize*). The coefficient of *ExtremeTemperature* is negative and statistically significant at the 1% level. It is consistent with our baseline result in Section 3.1, indicating that our main result is robust and concrete. The coefficient of the interaction term, ExtremeTemperature * LogFirmSize, is positive and significant at the 1% level. In the full specification in Column 4, the coefficient of ExtremeTemperature * LogFirmSize is 0.451. It indicates that a one-standard-deviation reduction in firm size from the sample mean is associated with a 0.451 increase in the temperature-return relation. The economic significance is large as the coefficient of ExtremeTemperature * LogFirmSize is equal to 83.8% of the coefficient of the basic term, *ExtremeTemperature*.

In Panel B, we focus on the impact of normalized firm age on the temperature-return relation. We follow Baker and Wurgler [24] and measure firm age as the natural logarithm of the number of years as the firm’s first appearance in the dataset, measured to the current month (*LogFirmAge*). Consistently, the coefficient of *ExtremeTemperature* is negative, significant at the 1% level across all specifications. As for the interaction term, ExtremeTemperature * LogFirmAge, we find the coefficient positive in four columns. Specifically, we find that the coefficient of ExtremeTemperature * LogFirmAge equals 0.461 in Column 4. It implies that a one-standard-deviation reduction in firm age from the sample mean will lead to a 0.461 increase in the magnitude of the temperature-return relation, which is about 70.0% of the coefficient of *ExtremeTemperature*.

In Panel C, we explore whether normalized volatility accentuates the link between extreme temperatures and stock returns. In the spirit of Baker and Wurgler [24], we measure volatility as the standard deviation of monthly returns over the past 12 months (*Volatility*). Not surprisingly, the coefficient of *ExtremeTemperature* is still negative and statistically significant. Besides, we observe a negative coefficient of ExtremeTemperature * Volatility. In the saturated specification from Column 4, the coefficient of ExtremeTemperature * Volatility equals −0.280, statistically significant at the 1% level. It suggests that a one-standard-deviation increase in volatility from the sample mean will increase the magnitude of the relation between extreme temperature and stock return by 0.280.

Overall, our findings suggest consistent evidence with Baker and Wurgler [24] regarding firm size, age, and volatility. Specifically, the link between extreme temperatures and the stock returns is more pronounced in smaller, younger, and more volatile firms.

#### 3.2.2. Profitability Characteristics

The second category of characteristics suggested by Baker and Wurgler [24] is profitability. We next investigate whether the profitability moderates the impact of extreme temperatures on the stock returns. We use ROE (return on equity) and ROA (return on asset) as two proxies for a firm’s profitability and construct the interaction terms.

The results are shown in Table 5. As expected, the coefficient of *ExtremeTemperature* is negative and statistically significant at the 1% level in Panels A and B. In Panel A, we use normalized *ROE*, which equals earnings divided by book equity in the recent fiscal year, to construct the interaction term. We find that the coefficient of ExtremeTemperature * ROE is positive, significant at the 1% level across all specifications. Specifically, the coefficient of ExtremeTemperature * ROE is 0.380 in Column 4. It indicates that a one-standard-deviation reduction in *ROE* from the sample mean is associated with a 0.380 increase in the magnitude of the temperature-return relation, or 73.1% of the coefficient of *ExtremeTemperature*.

In Panel B, we use normalized *ROA*, which equals earnings divided by assets, in the recent fiscal year, as an alternative proxy for profitability. The relation between ExtremeTemperature * ROA and *Ret_Abnormal* is positive and statistically significant in four columns. For example, the coefficient of the interaction term, ExtremeTemperature * ROA, equals 0.202 in Column 4, which is 39.8% of the coefficient of *ExtremeTemperature*. It indicates that a one-standard-deviation reduction in ROA from the sample mean will lead to a 0.202 increase in the link between extreme temperatures and stock returns. 

Taken together, we find that the relation between extreme temperatures and stock returns is more pronounced in firms with lower profitability. It is intuitive as less profitable firms are harder to value and arbitrage, exposing them more to mood fluctuations.

#### 3.2.3. Asset Tangibility Characteristics

Then, we aim to explore whether asset tangibility will influence the temperature-return relation. According to Baker and Wurgler [24], we construct the fixed asset ratio (*FixedAssets_ratio*) and R&D ratio (*RD_ratio*) as proxies for asset tangibility. Specifically, *FixedAssets_ratio* equals the fixed assets over total assets in the recent fiscal year, and *RD_ratio* equals the research and development expense over assets in the recent fiscal year.

Results are presented in Table 6. In Panel A, we use the normalized fixed assets to total assets to measure asset tangibility and construct the interaction term. Results show that the coefficient of ExtremeTemperature * FixedAssets_ratio is positive and statistically significant across all specifications. Specifically, the coefficient of ExtremeTemperature * FixedAssets_ratio in Column 4 equals 0.138, significant at the 5% level. It indicates that a one-standard-deviation reduction in fixed assets from the sample mean will lead to a 0.138 increase in the relation between extreme temperature and stock return. The coefficient of ExtremeTemperature * FixedAssets_ratio equals about 26.3% of the coefficient of *ExtremeTemperature*.

In Panel B, we calculate the normalized R&D ratio, an inverse proxy for asset tangibility. We observe a negative coefficient of the interaction term, ExtremeTemperature * RD_ratio, which is statistically significant. Specifically, we find that the coefficient of ExtremeTemperature * RD_ratio in Column 4 equals −0.213. It indicates that a one-standard-deviation increase in R&D ratio from the sample mean will lead to a 0.213 increase in the magnitude of the temperature-return relation. It also equals about 46.1% of the coefficient of *ExtremeTemperature*, indicating that the result is economically meaningful.

Collectively, our results suggest that firms with more intangible assets are more sensitive to fluctuations in mood. It is reasonable because firms with more intangible assets are difficult to value [63,64]. Thus, those firms are more likely to be influenced by mood. 

#### 3.2.4. Putting All the Firm Characteristics into an Overarching Model

Finally, we simultaneously include all the interaction terms in the regression models to see which factor is dominant. The results are reported in Table 7. For the size and age characteristics, we find that the coefficients of ExtremeTemperature * LogFirmSize, ExtremeTemperature * LogFirmAge, and ExtremeTemperature * Volatility are still significant and in the same direction as separately included, indicating that the firm size, firm age, and volatility perform well in our regression model.

For profitability characteristics, we find that the coefficient of ExtremeTemperature * ROE is significantly positive and consistent with the results in Table 5. The coefficient of ExtremeTemperature * ROA changes, presumably because the effect of *ROA* is absorbed by *ROE*. Nevertheless, the positive significance of ExtremeTemperature * ROE can also show that the firm’s profitability plays a role in influencing the relation between extreme temperature and stock performance.

For asset tangibility characteristics, the results show that the coefficient of ExtremeTemperature * FixedAssets_ratio becomes insignificant. As *RD_ratio* and *FixedAssets_ratio* are both proxies for asset tangibility, the insignificance of ExtremeTemperature * FixedAssets_ratio might be because the effect of *FixedAssets_ratio* is absorbed by *RD_ratio*. Despite the insignificance of ExtremeTemperature * FixedAssets_ratio, the coefficient of ExtremeTemperature * RD_ratio is still significantly negative and consistent with the results in Table 6. It suggests that asset tangibility affects our baseline finding. Overall, the three aspects of firm characteristics we consider in this section are all worthwhile in our empirical framework.

### 3.3. Are There Any Other Mediators Besides Mood?

In this section, we test whether extreme temperature affects firm’s stock returns through other mediators besides mood. Motivated by the previous literature on the effect of temperature on productivity [25,35], we first study whether productivity is a mediator. Then, we focus on the temperature-induced disasters and test whether disasters mediate the relation between extreme temperature and firm’s stock returns.

#### 3.3.1. Does Extreme Temperature Affect Firms’ Productivity?

It could be possible that extreme temperatures affect firms’ fundamental values by reducing labor productivity, thereby affecting the stock performance. If this is the case, we would expect to see a negative relation between extreme temperatures and productivity. Thus, we conduct the following regression model:

(7) $$FirmProductivity_{i,t}=\alpha +\beta_{1}\times ExtremeTemperature_{i,t}+\sum (\gamma_{n}\times Controls_{n,i,t})+\varepsilon_{i,t}$$

where $FirmProductivity_{i,t}$ is firm *i*’s productivity in month *t*. In the spirit of Addoum, Ng, and Ortiz-Bobea [25], we focus on two measures. First, we measure the firm’s operating performance as the natural logarithm of firm *i*’s sales during the year of month *t* (*LogSales*). Second, we measure labor productivity as the ratio of firm *i*’s sales to the number of employees during the year of month *t* (*WorkerProductivity*). $Controls_{i,t}$ are control variables, including lagged independent variables and other weather condition controls defined in Table 1. We add lagged *LogSales* of firm *i* in month *t*−1 (*Lagged_LogSales*) in Columns 1 and 2, while we include lagged *WorkerProductivity* of firm *i* in month *t*−1 (*Lagged_WorkerProductivity*) in Columns 3 and 4.

The regression results are reported in Table 8. We find no significant relation between extreme temperatures and sales in Columns 1 and 2. When we use worker productivity as the dependent variable, the results are similar and insignificant. These findings indicate that the inverse effects of extreme temperatures on stock returns are not caused by the reduced local labor productivity. The link is more presumably explained by the temperature-induced depression mood.

#### 3.3.2. Do Disasters Induced by Extreme Temperatures Provide Profit-Making Opportunities?

Higher exposure to extreme temperatures is related to a higher probability of disasters, such as windstorms or snowstorms. As the disasters devastate infrastructure, steel and construction material companies might have more profit-making opportunities to benefit from rebuilding the city’s infrastructure. In this case, extreme temperatures might have a positive effect on stock returns for these firms.

Therefore, we divide the firms into two groups (steel and construction firms, other firms) and replicate the regression model based on Equation (6) separately for the two groups. We define the firms that belong to the steelmaking industry, real estate industry, and civil engineering industry as steel and construction firms. Industries are defined using the China Securities Regulatory Commission classification method. Results are reported in Table 9. In Panel A, the impact of extreme temperatures on steel and construction firms’ stock performance is not significant across all columns. One possible explanation is that the result we find is a balance or combination of mood and profitability. On the one hand, extreme temperatures are associated with the depressed emotions of investors, resulting in lower stock returns. On the other hand, extreme temperature increases firms’ future values by providing profitable opportunities, shown as higher stock returns. Hence, the insignificant relation between extreme temperatures and stock returns in steel and construction industries seems reasonable, which is a trade-off between the two competing effects. In Panel B, we find a significantly negative relation between extreme temperatures and stock performance for firms other than construction firms, supporting that our main finding holds robust after considering the temperature-induced disaster effect.

### 3.4. Is the Impact of Extreme Temperature Driven by a Specific Sample?

#### 3.4.1. Excluding Agricultural Firms

One concern is that our main finding might only exist in agricultural firms. Specifically, extreme temperatures lessen the local crop yields, which leads to low stock returns. To mitigate this concern, we drop all agricultural firms in this section and repeat our analysis.

The results are reported in Table 10. The coefficient of *ExtremeTemperature* is negative and statistically significant at the 1% level in four columns. Specifically, the coefficient of *ExtremeTemperature* varies from −0.695 to −0.465. After excluding agricultural firms, the empirical results are robust. We find no significant changes in magnitude and significance. Overall, our results are not merely driven by agricultural firms.

#### 3.4.2. Excluding Months with Major Events

Another concern is that our results might be driven by events. For example, a negative earnings announcement may lead to negative stock performance. Besides, the market may also react negatively to the M&A announcement [65]. To rule out this concern, we exclude event months and repeat our baseline analysis.

Results are reported in Table 11. In Panel A, we exclude months in which firms issue quarterly reports, semi-annual reports, or annual reports. The coefficient of *ExtremeTemperature* is negative and statistically significant across all specifications. Specifically, the coefficient of *ExtremeTemperature* in Column 4 is −0.320, significant at the 5% level. In Panel B, we exclude months in which firms issue M&A announcements or will issue M&A announcements in the subsequent half a year. The relation between *ExtremeTemperature* and *Ret_Abnormal* is negative, significant at the 1% level. Specifically, the coefficient of *ExtremeTemperature* equals −0.489, which is statistically significant. Overall, our results are robust. The main finding is not merely driven by large events, such as quarterly reports and M&A announcements.

### 3.5. Robust Check for Alternative Specifications

#### 3.5.1. Alternative Approach to Match Firm’s Location with Weather Stations

In this section, we follow Goetzmann, Kim, Kumar, and Wang [15] to match all weather stations within a 50 km radius of the firm’s location for robustness check. We first calculate the proportion of days with extreme temperatures in a month for each related station and denote this variable as *ExtremeTemperatureOrg.* We then use the synthetic control method where a hypothetical weather station is constructed by using geometric weights of all weather stations within the radius. Specifically, we use 50 minus the distance between the firm’s location and weather stations as the weights to calculate the weighted average *ExtremeTemperatureOrg*. Thus, closer stations are given a higher weighting. We denote this robust measure as *ExtremeTemperatureVw* and replicate the baseline regression model based on Equation (6). The results are reported in Table 12. The coefficients of *ExtremeTemperatureVw* are significant across all specifications and similar to the coefficients of *ExtremeTemperature* in Table 3. It suggests that this limitation does not matter in our analyses.

#### 3.5.2. Calculating Abnormal Returns by Using Alternative Formation Periods

To verify the robustness, we use different formation periods to estimate the expected stock return and repeat our baseline regression. The formation period of βs is generally 24 to 60 months [30]. Hence, we use stock returns in the past 36, 48, and 60 months in this section to estimate the expected return and construct the abnormal stock return.

The results are reported in Table 13. In Panel A, the estimation window is 36 months. The coefficient of *ExtremeTemperature* is negative and statistically significant at the 1% level. The robustness is formally validated in Column 4. The coefficient of *ExtremeTemperature* is −0.380, statistically significant at the 1% level. In Panel B, we use returns in the past 48 months to estimate the βs. The result is robust as the relation between *ExtremeTemperature* and *Ret_Abnormal* is still negative and statistically significant. Specifically, the coefficient of *ExtremeTemperature* in Column 4 is −0.297. In Panel C, the estimation window is 60 months. We observe a negative link between *ExtremeTemperature* and *Ret_Abnormal*, which is statistically significant. The coefficient of *ExtremeTemperature* in Column 4, including all controls, is −0.276. Collectively, our findings are not sensitive to the estimation windows. The results show the robustness as the sign and significance are quite unchanged. 

#### 3.5.3. Alternative Asset Pricing Models

This section uses the FF3 and FF5 models to estimate factor loadings on risk premiums for robustness. The methods of estimating factor loadings based on FF3 and FF5 models are similar to estimating the loading on the market factor of *Ret_Abnormal*. We use new estimated factor loadings to calculate expected and abnormal returns and then repeat the baseline regression.

We report the results in Table 14. In Panel A, abnormal return is calculated based on the FF3 model. The coefficient of *ExtremeTemperature* is statistically negative at the 1% level across all specifications. Specifically, the coefficients of *ExtremeTemperature* vary from −0.464 to −0.201, indicating that a larger proportion of extreme ambient temperatures in a month will lead to a more negative stock reaction. In Panel B, we calculate the abnormal return based on the FF5 model. Consistently, we find a robust link between *ExtremeTemperature* and *Ret_Abnormal* (FF5), which is negative and statistically significant. Taken together, the results in Table 14 suggest that our main finding is not sensitive to the asset pricing models, and investors react negatively to firms when experiencing unpleasant ambient temperatures.

#### 3.5.4. Alternative Measures for Extreme Temperature 

Finally, we explore whether extremely hot and cold environments affect mood differently. We split *ExtremeTemperature* into two parts, extremely hot temperature (*ExtremeTemperature_Hot*) and extremely cold temperature (*ExtremeTemperature_Cold*), and repeat our baseline regression. *ExtremeTemperature_Hot* (*ExtremeTemperature_Cold*) denotes the fraction of days with extreme temperature above 30 °C (below 0 °C) in each trading month for each firm’s headquarter address.

Results are shown in Panel A of Table 15. We include *ExtremeTemperature_Hot* and *ExtremeTemperature_Cold* into one regression simultaneously. Results show that both *ExtremeTemperature_Hot* and *ExtremeTemperature_Cold* are negative and statistically significant across all specifications. In our favored specification from Column 4, the coefficient of *ExtremeTemperature_Hot* is −0.544, and the coefficient of *ExtremeTemperature_Cold* is −0.452. We observe no significant difference between the two coefficients regarding the sign, significance, and magnitude. It indicates that the impact of extreme temperature does not only exist in hot or cold environments. Instead, both extremely hot and cold environments have a significant impact on investors’ mood.

In Panel B, we calculate the average temperature deviation from the optimum ambient temperature in a month, *ExtremeTemperature_Conti*. We find that the coefficient of *ExtremeTemperature_Conti* is also negative, statistically significant at the 1% level in four columns. The result indicates that a larger deviation from the most comfortable ambient temperature is associated with a more depressed mood, which will lead to lower stock returns.

## 4. Discussion

Inspired by the increasing extreme temperature cases, we explore how exposures to extreme temperatures affect investors’ mood and then influence stock return. We find that more exposures to extreme temperatures are associated with lower stock returns. This result is more pronounced in hard-to-value firms, such as firms with smaller sizes, younger ages, higher volatility, lower profitability, and higher intangible asset ratios. The concrete evidence suggests that mood plays a role.

From the perspective of mood, Cao and Wei [10] were the first to explore how temperature affects the stock market. Using nine international stock indices from 1962 to 2001, they find a negative relation between the temperatures of exchanges’ locations and index returns. Similarly, Dong and Tremblay [46] also use international indices to explore the effect of weather conditions on market returns. Instead of analyzing at an aggregate level from a relatively macro perspective, we study the temperature of each listed firm’s location and employ a firm-level analysis. Moreover, Cao and Wei [10] and Dong and Tremblay [46] study the linear correlation between temperature and index returns. However, the relation between temperature and mood might follow a U-shape; that is, extremely cold and hot weather might both cause depression. Therefore, instead of directly using temperature, we analyze how firm-level stock returns change with extreme temperature exposures. Due to the lack of outdoor time data, we cannot investigate whether the duration of outdoor exposures has a significant impact on people’s mood like Dong and Tremblay [46]. We believe it could be an interesting extension for our study. 

Besides, Makridis [66] uses survey data about individuals’ attitudes over future economic activity in the US. They focus on how extreme temperature affects individuals’ beliefs on the future economy. Unlike their focus, we investigate the effect of extreme temperature on firm’s stock performance. Furthermore, we also discuss the heterogeneity in the temperature-return relation based on firms’ characteristics, such as firm size and age.

From the perspective of climate risk, Balvers, Du, and Zhao [11] analyze the temperature effect in an Arbitrage Pricing Theory model in the US stock market and find that temperature shocks are a systematic risk factor with a significantly negative risk premium. Similarly, Kumar et al. [67] also explore the link between the exposures to climate risk and stock performance and find that higher climate sensitivity will lead to lower stock returns. Our paper differs from them in the following ways. First, we analyze the firm-specific temperature while Balvers, Du, and Zhao [11] analyze the time-series temperature on an aggregate level. Second, we focus on investor mood induced by extreme temperature. Instead, Balvers, Du, and Zhao [11] and Kumar, Xin, and Zhang [67] use temperature to measure climate risk. 

Our tests also provide some practical implications. Due to various reasons, extreme weather is becoming increasingly frequent and severe, and many countries have suffered enormous losses. Extreme weather leads to substantial impacts, such as loss of life, damages to agricultural production, and economic effects [68]. Specifically, China has witnessed natural disasters featured by flooding, typhoon, drought, earthquake, geological disasters, and so on in 2019. Among them, there were 2345 forest fires, with 8 major fires and 1 conflagration, and 20 earthquakes registering above magnitude 5 [69]. The total direct economic loss of natural disasters was about 327.09 billion RMB [70]. Hence, it is important to implement regulations to improve environmental conditions to avoid unexpected capital loss.

Besides those macro-level impacts, the ambient environment has long been an important determinant of daily mood [71], significantly influencing one’s decisions. Notably, depression is not simply a kind of emotion; it is also related to mental health problems and illness [72]. Extreme temperatures can be even fatal to people’s health [73,74,75]. Hence, the linkage between climate conditions and mental health is increasingly being discussed [76]. Hansen et al. [77] explore the impact of extremely hot temperatures and find that exposures to extreme heat cause a noticeable risk to the health and well-being of the mentally ill. Burke, González, Baylis, Heft-Neal, Baysan, Basu, and Hsiang [60] analyze the depressive language in social media updates and find that higher temperatures deteriorate mental well-being. Besides, suicide rates rise by 0.7% in US counties and 2.1% in Mexican municipalities when the monthly average temperature rises by 1 °C. This effect is significant and indifferent in hotter and colder regions and will not decay over time. Extreme temperatures can also cause more conflicts, injuries, and mortality [78]. Given the large potential change in temperatures in the following decades [78,79,80], the temperature-induced pessimism or even mental illness is expected to be more widespread, and its impact tends to be more pronounced. Hence, care of the depressed emotions needs to be addressed to avoid an increase in severe mental illness.

Overall, our findings imply the depression of investors induced by extreme temperatures on average. It is meaningful and insightful to figure out the impact of extreme temperatures from the perspectives of intensity, frequency, and duration. However, due to data limitation, we have no access to describe the duration and acceleration of extreme temperatures and link them to the stock performance from a relatively micro (intra-day) perspective. We leave this interesting question for future work.

## 5. Conclusions

This paper demonstrates the negative link between extreme temperatures and stock returns, which can be explained by the depressed weather-induced mood. This result is more pronounced in firms that tend to be more influenced by investor sentiments, such as firms with smaller sizes, younger ages, higher volatility, lower profitability, and higher intangible asset ratios. This evidence supports the view that mood has an impact on the financial market. 

Additionally, our findings are not merely mediated by reduced labor productivity, potential climate disasters, or driven by agricultural firms and major events, and are also not sensitive to matching method, formation periods, and asset pricing models. Finally, we find that exposure to both extremely high and low temperatures will induce a low stock return without significant difference. 

## Figures and Tables

**Table 1:** Variable definitions.

Variables	Description
ExtremeTemperature	*ExtremeTemperature* is the fraction of days with extreme temperature in each trading month for each firm’s headquarter address. Following Addoum, Ng, and Ortiz-Bobea [25], we define a day as an extreme temperature day if the temperature exceeds an upper limit of 30 °C or drops below 0 °C.
SAD	In the spirit of Kamstra, Kramer, and Levi [48], we measure *SAD* as the average night duration (in hours) minus 12 for each firm’s headquarter address. To consider the night duration in all seasons, we do not replace the values with zero in spring and summer.
Sunshine	*Sunshine* is the average sunshine duration (sunshine hours per day/24) in each month for each firm’s headquarter address.
Rain	*Rain* is the fraction of rainy days in each month for each firm’s headquarter address.
Wind	*Wind* equals the fraction of strong wind days in each month for each firm’s headquarter address. Following the previous literature, we define a day as a strong windy day if the daily wind speed is higher than 5 km/h.
Humidity	*Humidity* is the average relative humidity in each month for each firm’s headquarter address.
AirPressure	*AirPressure* is the average air pressure (in 100 kPa) in each month for each firm’s headquarter address.

**Table 2:** Summary statistics of variables.

	N	Mean	SD	P5	P25	P50	P75	P95
Ret_Abnormal (%)	294,795	0.404	11.186	−15.994	−6.030	−0.781	5.592	20.768
ExtremeTemperature	359,561	0.361	0.351	0.000	0.032	0.233	0.700	1.000
SAD	359,628	−0.117	1.522	−2.451	−1.407	−0.008	1.158	2.212
Sunshine	358,536	0.219	0.081	0.082	0.163	0.219	0.276	0.353
Rain	359,561	0.318	0.178	0.033	0.179	0.300	0.452	0.633
Wind	359,561	0.784	0.215	0.300	0.677	0.867	0.935	1.000
Humidity	358,554	0.699	0.122	0.440	0.636	0.726	0.789	0.853
AirPressure	359,230	0.995	0.041	0.897	0.998	1.007	1.015	1.025

**Table 3:** The impact of extreme temperature on firm-specific stock return.

Dependent =	Ret_Abnormal			
	(1)	(2)	(3)	(4)
ExtremeTemperature	−0.669 ***	−0.695 ***	−0.468 ***	−0.500 ***
	(0.074)	(0.106)	(0.073)	(0.107)
Lagged_Ret_Abnormal		−0.052 ***		−0.069 ***
		(0.002)		(0.002)
SAD		0.275 ***		0.175 **
		(0.076)		(0.076)
Sunshine		0.385		2.252 ***
		(0.547)		(0.551)
Rain		−1.118 ***		−1.207 ***
		(0.213)		(0.212)
Wind		−0.402 **		−0.773 ***
		(0.163)		(0.153)
Humidity		3.623 ***		4.589 ***
		(0.395)		(0.398)
AirPressure		−4.854 **		−5.061 **
		(2.414)		(2.049)
Stock FEs?	Yes	Yes	Yes	Yes
Month FEs?	Yes	Yes	Yes	Yes
Year FEs?	No	No	Yes	Yes
Number of obs.	294,707	288,558	294,707	288,558
Adjusted R-squared	0.015	0.019	0.040	0.046

Note: **, and *** indicate statistical significance at 10%, 5% and 1% levels, respectively.

**Table 4:** The impact of extreme temperature across size and age characteristics.

Dependent =	Ret_Abnormal			
	(1)	(2)	(3)	(4)
**Panel A: Firm size**				
ExtremeTemperature	−0.651 ***	−0.610 ***	−0.499 ***	−0.538 ***
	(0.074)	(0.107)	(0.074)	(0.107)
ExtremeTemperature * LogFirmSize	0.430 ***	0.430 ***	0.452 ***	0.451 ***
	(0.066)	(0.068)	(0.066)	(0.068)
LogFirmSize	−1.613 ***	−1.626 ***	−1.284 ***	−1.218 ***
	(0.047)	(0.049)	(0.063)	(0.065)
Number of obs.	294,375	288,238	294,375	288,238
Adjusted R-squared	0.020	0.024	0.037	0.042
**Panel B: Firm age**				
ExtremeTemperature	−0.780 ***	−0.732 ***	−0.619 ***	−0.659 ***
	(0.080)	(0.111)	(0.079)	(0.111)
ExtremeTemperature * LogFirmAge	0.484 ***	0.474 ***	0.482 ***	0.461 ***
	(0.085)	(0.089)	(0.084)	(0.089)
LogFirmAge	−1.846 ***	−1.977 ***	−1.166 ***	−1.268 ***
	(0.059)	(0.063)	(0.118)	(0.127)
Number of obs.	294,707	288,558	294,707	288,558
Adjusted R-squared	0.018	0.023	0.035	0.041
**Panel C: Volatility**				
ExtremeTemperature	−0.661 ***	−0.694 ***	−0.501 ***	−0.550 ***
	(0.075)	(0.108)	(0.074)	(0.108)
ExtremeTemperature * Volatility	−0.069	−0.089	−0.269 ***	−0.280 ***
	(0.081)	(0.083)	(0.082)	(0.085)
Volatility	0.242 ***	0.323 ***	−0.535 ***	−0.421 ***
	(0.037)	(0.039)	(0.042)	(0.044)
Number of obs.	294,652	288,532	294,652	288,532
Adjusted R-squared	0.015	0.020	0.037	0.042
**Panel A–C:**				
Controls?	No	Yes	No	Yes
Stock FEs?	Yes	Yes	Yes	Yes
Month FEs?	Yes	Yes	Yes	Yes
Year FEs?	No	No	Yes	Yes

Note: *** indicate statistical significance at 10%, 5% and 1% levels, respectively.

**Table 5:** The impact of extreme temperature across profitability characteristics.

Dependent =	Ret_Abnormal			
	(1)	(2)	(3)	(4)
**Panel A: ROE**				
ExtremeTemperature	−0.678 ***	−0.702 ***	−0.481 ***	−0.520 ***
	(0.074)	(0.106)	(0.073)	(0.106)
ExtremeTemperature * ROE	0.370 ***	0.403 ***	0.342 ***	0.380 ***
	(0.086)	(0.087)	(0.085)	(0.087)
ROE	−0.147 ***	−0.135 ***	−0.158 ***	−0.155 ***
	(0.036)	(0.037)	(0.034)	(0.035)
Number of obs.	294,688	288,540	294,688	288,540
Adjusted R-squared	0.015	0.019	0.035	0.041
**Panel B: ROA**				
ExtremeTemperature	−0.665 ***	−0.688 ***	−0.468 ***	−0.507 ***
	(0.074)	(0.106)	(0.073)	(0.106)
ExtremeTemperature * ROA	0.165 **	0.210 ***	0.155 **	0.202 ***
	(0.075)	(0.076)	(0.073)	(0.075)
ROA	−0.105 ***	−0.090 **	−0.139 ***	−0.132 ***
	(0.037)	(0.037)	(0.034)	(0.035)
Number of obs.	294,688	288,540	294,688	288,540
Adjusted R-squared	0.015	0.019	0.035	0.041
**Panel A–B:**				
Controls?	No	Yes	No	Yes
Stock FEs?	Yes	Yes	Yes	Yes
Month FEs?	Yes	Yes	Yes	Yes
Year FEs?	No	No	Yes	Yes

Note: **, and *** indicate statistical significance at 10%, 5% and 1% levels, respectively.

**Table 6:** The impact of extreme temperature across asset tangibility characteristics.

Dependent =	Ret_Abnormal			
	(1)	(2)	(3)	(4)
**Panel A: Fixed assets**				
ExtremeTemperature	−0.667 ***	−0.690 ***	−0.478 ***	−0.524 ***
	(0.074)	(0.106)	(0.073)	(0.106)
ExtremeTemperature * FixedAssets_ratio	0.159 **	0.147 **	0.143 **	0.138 **
	(0.065)	(0.066)	(0.064)	(0.066)
FixedAssets_ratio	0.614 ***	0.577 ***	0.287 ***	0.246 ***
	(0.055)	(0.056)	(0.049)	(0.050)
Number of obs.	294,688	288,540	294,688	288,540
Adjusted R-squared	0.016	0.020	0.035	0.041
**Panel B: Research and development expense**				
ExtremeTemperature	−0.562 ***	−0.627 ***	−0.410 ***	−0.462 ***
	(0.086)	(0.123)	(0.085)	(0.123)
ExtremeTemperature * RD_ratio	−0.268 ***	−0.248 ***	−0.224 ***	−0.213 **
	(0.087)	(0.090)	(0.085)	(0.089)
RD_ratio	−0.164 ***	−0.178 ***	0.016	0.013
	(0.056)	(0.057)	(0.050)	(0.051)
Number of obs.	228,449	224,098	228,449	224,098
Adjusted R-squared	0.015	0.020	0.033	0.040
**Panel A–B:**				
Controls?	No	Yes	No	Yes
Stock FEs?	Yes	Yes	Yes	Yes
Month FEs?	Yes	Yes	Yes	Yes
Year FEs?	No	No	Yes	Yes

Note: **, and *** indicate statistical significance at 10%, 5% and 1% levels, respectively.

**Table 7:** Overarching regression models with the inclusion of all interaction terms.

Dependent =	Ret_Abnormal			
	(1)	(2)	(3)	(4)
ExtremeTemperature	−0.664 ***	−0.656 ***	−0.532 ***	−0.569 ***
	(0.093)	(0.129)	(0.093)	(0.130)
ExtremeTemperature * LogFirmSize	0.252 ***	0.259 ***	0.238 ***	0.241 ***
	(0.080)	(0.083)	(0.080)	(0.083)
ExtremeTemperature * LogFirmAge	0.347 ***	0.325 ***	0.356 ***	0.316 ***
	(0.108)	(0.112)	(0.108)	(0.113)
ExtremeTemperature * Volatility	−0.158	−0.170 *	−0.330 ***	−0.337 ***
	(0.097)	(0.099)	(0.098)	(0.101)
ExtremeTemperature * ROE	0.796 ***	0.771 ***	0.789 ***	0.772 ***
	(0.214)	(0.216)	(0.215)	(0.218)
ExtremeTemperature * ROA	−0.408 **	−0.345 *	−0.399 **	−0.340 *
	(0.184)	(0.187)	(0.184)	(0.187)
ExtremeTemperature * FixedAssets_ratio	0.047	0.031	0.035	0.018
	(0.074)	(0.076)	(0.074)	(0.076)
ExtremeTemperature * RD_ratio	−0.231 ***	−0.214 **	−0.236 ***	−0.232 **
	(0.088)	(0.091)	(0.087)	(0.090)
LogFirmSize	−1.520 ***	−1.453 ***	−1.588 ***	−1.534 ***
	(0.077)	(0.078)	(0.084)	(0.085)
LogFirmAge	0.118	−0.087	−0.916 ***	−1.054 ***
	(0.103)	(0.107)	(0.158)	(0.169)
Volatility	−0.060	0.050	−0.606 ***	−0.457 ***
	(0.044)	(0.046)	(0.048)	(0.051)
ROE	−0.110	−0.118	−0.075	−0.088
	(0.083)	(0.084)	(0.082)	(0.083)
ROA	−0.010	−0.001	−0.033	−0.024
	(0.085)	(0.088)	(0.083)	(0.086)
FixedAssets_ratio	0.168 ***	0.126 **	0.157 ***	0.136 **
	(0.061)	(0.062)	(0.060)	(0.062)
RD_ratio	−0.033	−0.027	0.029	0.028
	(0.055)	(0.056)	(0.051)	(0.052)
Controls?	No	Yes	No	Yes
Stock FEs?	Yes	Yes	Yes	Yes
Month FEs?	Yes	Yes	Yes	Yes
Year FEs?	No	No	Yes	Yes
Number of obs.	228,165	223,848	228,165	223,848
Adjusted R-squared	0.018	0.023	0.037	0.043

Note: *, **, and *** indicate statistical significance at 10%, 5% and 1% levels, respectively.

**Table 8:** The impact of extreme temperature on firm’s productivity.

Dependent =	LogSales		WorkerProductivity	
	(1)	(2)	(1)	(2)
ExtremeTemperature	0.008	0.008	0.001	−0.001
	(0.006)	(0.007)	(0.001)	(0.002)
Lagged_LogSales		0.468 ***		
		(0.011)		
Lagged_WorkerProductivity				0.505 ***
				(0.021)
SAD		−0.005		0.001
		(0.004)		(0.001)
Sunshine		−0.009 ***		−0.001
		(0.003)		(0.001)
Rain		−0.015		−0.001
		(0.015)		(0.004)
Wind		0.030		0.019 **
		(0.035)		(0.008)
Humidity		−0.001 **		0.000
		(0.001)		(0.000)
AirPressure		0.001 *		−0.000
		(0.001)		(0.000)
Stock FEs?	Yes	Yes	Yes	Yes
Month FEs?	Yes	Yes	Yes	Yes
Year FEs?	Yes	Yes	Yes	Yes
Number of obs.	305,913	256,107	305,621	255,604
Adjusted R-squared	0.749	0.822	0.671	0.771

Note: *, **, and *** indicate statistical significance at 10%, 5% and 1% levels, respectively.

**Table 9:** The impact of extreme temperature: Steel and construction firms vs. other firms.

Dependent =	Ret_Abnormal			
	(1)	(2)	(3)	(4)
**Panel A: Steel and** **construction firms**				
ExtremeTemperature	1.704	−0.385	1.847	−0.225
	(2.332)	(0.479)	(2.348)	(0.480)
Number of obs.	28,040	27,646	28,040	27,646
Adjusted R-squared	−0.000	0.012	0.000	0.020
**Panel B: Other firms**				
ExtremeTemperature	−0.708 ***	−0.709 ***	−0.468 ***	−0.501 ***
	(0.102)	(0.121)	(0.101)	(0.120)
Number of obs.	266,667	260,912	266,667	260,912
Adjusted R-squared	0.009	0.017	0.024	0.038
**Panel A–B:**				
Controls?	No	Yes	No	Yes
Stock FEs?	Yes	Yes	Yes	Yes
Month FEs?	Yes	Yes	Yes	Yes
Year FEs?	No	No	Yes	Yes

Note: *** indicate statistical significance at 10%, 5% and 1% levels, respectively.

**Table 10:** Baseline regressions of excluding agriculture firms.

Dependent =	Ret_Abnormal			
	(1)	(2)	(3)	(4)
ExtremeTemperature	−0.665 ***	−0.695 ***	−0.465 ***	−0.507 ***
	(0.074)	(0.107)	(0.073)	(0.107)
Controls?	No	Yes	No	Yes
Stock FEs?	Yes	Yes	Yes	Yes
Month FEs?	Yes	Yes	Yes	Yes
Year FEs?	No	No	Yes	Yes
Number of obs.	290,388	284,316	290,388	284,316
Adjusted R-squared	0.015	0.019	0.035	0.041

Note: *** indicate statistical significance at 10%, 5% and 1% levels, respectively.

**Table 11:** Baseline regressions of excluding months with major events.

Dependent =	Ret_Abnormal			
	(1)	(2)	(3)	(4)
**Panel A: Ex months issuing reports**				
ExtremeTemperature	−0.653 ***	−0.552 ***	−0.403 ***	−0.320 **
	(0.084)	(0.127)	(0.083)	(0.127)
Number of obs.	209,873	205,266	209,873	205,266
Adjusted R-squared	0.017	0.024	0.044	0.052
**Panel B: Ex months subsequent M&A**				
ExtremeTemperature	−0.640 ***	−0.643 ***	−0.472 ***	−0.489 ***
	(0.075)	(0.109)	(0.074)	(0.109)
Number of obs.	275,459	270,491	275,459	270,491
Adjusted R-squared	0.015	0.019	0.033	0.039
**Panel A–B:**				
Controls?	No	Yes	No	Yes
Stock FEs?	Yes	Yes	Yes	Yes
Month FEs?	Yes	Yes	Yes	Yes
Year FEs?	No	No	Yes	Yes

Note: **, and *** indicate statistical significance at 10%, 5% and 1% levels, respectively.

**Table 12:** Matching each firm’s location with all weather stations within a radius.

Dependent =	Ret_Abnormal			
	(1)	(2)	(3)	(4)
ExtremeTemperatureVw	−0.603 ***	−0.611 ***	−0.398 ***	−0.404 ***
	(0.077)	(0.114)	(0.076)	(0.114)
Controls?	No	Yes	No	Yes
Stock FEs?	Yes	Yes	Yes	Yes
Month FEs?	Yes	Yes	Yes	Yes
Year FEs?	No	No	Yes	Yes
Number of obs.	270,416	264,716	270,416	264,716
Adjusted R-squared	0.015	0.020	0.040	0.047

Note: *** indicate statistical significance at 10%, 5% and 1% levels, respectively.

**Table 13:** Estimating expected returns by using alternative formation periods.

	(1)	(2)	(3)	(4)
**Panel A: Formation period is [−36, −1]**	**Dependent = Ret_Abnormal (3-years)**			
ExtremeTemperature	−0.548 ***	−0.522 ***	−0.372 ***	−0.380 ***
	(0.074)	(0.110)	(0.074)	(0.110)
Number of obs.	271,405	265,883	271,405	265,883
Adjusted R-squared	0.012	0.017	0.035	0.042
**Panel B: Formation period is [−48, −1]**	**Dependent = Ret_Abnormal (4-years)**			
ExtremeTemperature	−0.480 ***	−0.408 ***	−0.315 ***	−0.297 ***
	(0.075)	(0.112)	(0.075)	(0.112)
Number of obs.	251,899	246,844	251,899	246,844
Adjusted R-squared	0.011	0.016	0.032	0.039
**Panel C: Formation period is [−60, −1]**	**Dependent = Ret_Abnormal (5-years)**			
ExtremeTemperature	−0.465 ***	−0.359 ***	−0.318 ***	−0.276 **
	(0.076)	(0.114)	(0.075)	(0.114)
Number of obs.	233,628	229,018	233,628	229,018
Adjusted R-squared	0.012	0.018	0.030	0.037
**Panel A–C:**				
Controls?	No	Yes	No	Yes
Stock FEs?	Yes	Yes	Yes	Yes
Month FEs?	Yes	Yes	Yes	Yes
Year FEs?	No	No	Yes	Yes

Note: **, and *** indicate statistical significance at 10%, 5% and 1% levels, respectively.

**Table 14:** Estimating expected returns by using alternative asset pricing models.

	(1)	(2)	(3)	(4)
**Panel A: Fama–French three-factor model**	**Dependent = Ret_Abnormal (FF3)**			
ExtremeTemperature	−0.201 ***	−0.453 ***	−0.211 ***	−0.464 ***
	(0.076)	(0.109)	(0.076)	(0.110)
Number of obs.	294,707	288,558	294,707	288,558
Adjusted R-squared	0.000	0.003	0.005	0.008
**Panel B: Fama–French five-factor model**	**Dependent = Ret_Abnormal (FF5)**			
ExtremeTemperature	−0.168 **	−0.373 ***	−0.164 **	−0.365 ***
	(0.082)	(0.119)	(0.082)	(0.119)
Number of obs.	294,707	288,558	294,707	288,558
Adjusted R-squared	0.003	0.005	0.010	0.013
**Panel A–B:**				
Controls?	No	Yes	No	Yes
Stock FEs?	Yes	Yes	Yes	Yes
Month FEs?	Yes	Yes	Yes	Yes
Year FEs?	No	No	Yes	Yes

Note: **, and *** indicate statistical significance at 10%, 5% and 1% levels, respectively.

**Table 15:** Alternative specifications of extreme temperature.

Dependent =	Ret_Abnormal			
	(1)	(2)	(3)	(4)
**Panel A: Extremely hot and cold**				
ExtremeTemperature_Hot	−1.247 ***	−0.907 ***	−0.625 ***	−0.544 ***
	(0.144)	(0.162)	(0.142)	(0.163)
ExtremeTemperature_Cold	−0.269 **	−0.466 ***	−0.359 ***	−0.452 ***
	(0.111)	(0.169)	(0.110)	(0.170)
Number of obs.	294,707	288,558	294,707	288,558
Adjusted R-squared	0.015	0.019	0.040	0.046
**Panel B: Continuous extreme temperature**				
ExtremeTemperature_Conti	−0.049 ***	−0.109 ***	−0.047 ***	−0.107 ***
	(0.005)	(0.010)	(0.005)	(0.009)
Number of obs.	293,948	288,558	293,948	288,558
Adjusted R-squared	0.015	0.020	0.040	0.046
**Panel A–B:**				
Controls?	No	Yes	No	Yes
Stock FEs?	Yes	Yes	Yes	Yes
Month FEs?	Yes	Yes	Yes	Yes
Year FEs?	No	No	Yes	Yes

Note: **, and *** indicate statistical significance at 10%, 5% and 1% levels, respectively.

## Data Availability

The data analyzed for this study are publicly available at https://www.gtarsc.com/.
